# Field Susceptibility of Almond (*Prunus dulcis*) Cultivars to Red Leaf Blotch Caused by *Polystigma amygdalinum* in Apulia (Italy) and Influence of Environmental Conditions

**DOI:** 10.3390/plants15020188

**Published:** 2026-01-07

**Authors:** Pompea Gabriella Lucchese, Emanuele Chiaromonte, Donato Gerin, Angelo Agnusdei, Francesco Dalena, Davide Cornacchia, Davide Digiaro, Giuseppe Incampo, Davide Salamone, Pasquale Venerito, Francesco Faretra, Franco Nigro, Stefania Pollastro

**Affiliations:** 1 Department of Soil, Plant and Food Sciences, University of Bari Aldo Moro, Via Amendola 165/A, 70126 Bari, Italy; pompea.lucchese@uniba.it (P.G.L.); emanuele.chiaromonte@uniba.it (E.C.); angelo.agnusdei@uniba.it (A.A.); davide.cornacchia@uniba.it (D.C.); giuseppeincampo99@gmail.com (G.I.); francesco.faretra@uniba.it (F.F.); franco.nigro@uniba.it (F.N.);; 2 Centro di Ricerca, Sperimentazione e Formazione in Agricoltura “Basile Caramia”, Via Cisternino 281, 70010 Locorotondo, Italy; 3 Agrimeca Grape and Fruit Consulting, Via J. F. Kennedy, 6, 70038 Terlizzi, Italy; d.digiaro@agrimeca.eu

**Keywords:** varietal clustering, environmental factors, qPCR validation, germplasm biodiversity

## Abstract

*Polystigma amygdalinum* the causal agent of Red Leaf Blotch (RLB), is responsible for one of the most important foliar diseases affecting almond [*Prunus dulcis* (Miller) D.A. Webb] in the Mediterranean Basin and the Middle East. The study is aimed at improving knowledge on RLB epidemiology and the role of environmental conditions in disease development. Field monitoring was conducted from 2022 to 2025 in three almond orchards located in Apulia (southern Italy) and characterized by different microclimatic conditions. A total of 39 cultivars, including Apulian local germplasm and international cultivars (‘Belona’, ‘Genco’, ‘Guara’, ‘Ferragnès’, ‘Filippo Ceo’, ‘Lauranne^®^ Avijor’, ‘Soleta’, and ‘Supernova’), were evaluated. Symptoms occurred from late spring to summer, resulting particularly severe on ‘Guara’ and ‘Lauranne^®^ Avijor’, whereas ‘Belona’, ‘Ferragnès’, ‘Genco’, and ‘Supernova’ exhibited the highest tolerance. To our knowledge, this is also the first report of RLB tolerance by ‘Filippo Ceo’, ‘Ficarazza’, ‘Centopezze’, and ‘Rachele piccola’ representing potential genetic resources for breeding programs. Moreover, these findings reinforced previous observations proving that RLB was less severe on medium-late and late cultivars. Disease incidence varied significantly among sites and years and was strongly associated with increased rainfall, higher relative humidity, and mild temperatures recorded in November, influencing disease occurrence in the following growing season. *P. amygdalinum* was consistently detected by qPCR in all RLB-affected tissues and, in some cases, from mixed early RLB + *Pseudomonas*-like symptoms. From some leaves with early RLB symptoms, *P. amygdalinum* was also successfully isolated in pure culture. Overall, our results provide clear evidence that *P. amygdalinum* is the sole fungal pathogen consistently associated with typical RLB symptoms in Apulia (southern Italy) and highlight important cultivar-dependent differences. Its frequent molecular detection in leaves showing atypical or mixed symptoms suggests unresolved epidemiological aspects requiring further investigation.

## 1. Introduction

Red Leaf Blotch (RLB) is one of the most significant foliar diseases affecting almond [*Prunus dulcis* (Miller) D.A. Webb] [1]. RLB is caused by *Polystigma amygdalinum* P.F. Cannon, a hemibiotrophic ascomycete strictly specialized on almond and notoriously difficult to culture in vitro [2,3,4]. The pathogen was initially described in Italy as *Septoria rubra* var. *amygdalina* [3], subsequently re-reported in the 1970s [5] and later reclassified within the genus *Polystigma* as *P. ochraceum* or *P. rubrum* [5].

Detailed investigations of the pathogen’s life cycle and epidemiology indicate that RLB is a monocyclic disease confined to foliage [2]. Ascospores produced in perithecia formed on infected leaves fallen during the previous season constitute the primary inoculum [6]. Infections occur on young, emerging leaves shortly after petal fall, particularly when spring rainfall events coincide with periods of high leaf susceptibility [7,8]. Early symptoms manifest as pale yellowish spots or blotches on both leaf surfaces. These lesions gradually enlarge, reaching up to 2 cm in diameter, and develop a yellow–orange halo surrounding a reddish-brown centre. At advanced stages, leaves turn blackish-brown, become necrotic, curl, and prematurely abscise [9], ultimately leading to reduced photosynthetic capacity and yield losses [10]. Lesions consist of fungal stromata embedded within host tissues [11] and may vary in shape and size, typically ranging from circular to elliptical, with final diameters of 11–22 mm and generally not exceeding 25 mm. Under conducive environmental conditions, severe premature defoliation may occur [9].

It is an endemic pathology widely distributed across Europe and Asia [5,6,7], where it is considered a disease of high economic relevance [3,12,13]. Recently, RLB has also been introduced into California [6]. In recent years, substantial increases in disease incidence have been reported in Spain [11,14], particularly in southern regions where climatic conditions are highly favourable for pathogen development [15] and where newly planted cultivars tend to be more susceptible.

In southern Italy (Siracusa, Sicily), ref. [16] reported severe RLB infections during summer 2009 in two almond orchards, demonstrating that the cultivars ‘Tuono’ and ‘Supernova’ were more susceptible than ‘Ferragnès’.

Multiple studies have highlighted the strong relationship between climatic variables and RLB development. In Spain, the potential ascospore release period extends from February to July, peaking in March–April [13]. A similar March–May peak in ascospore dispersal was observed in France [17].

As a monocyclic disease, the agronomic practices are essentially focused at reducing the primary inoculum represented by the ascospores dispersed by the perithecia produced in the fallen affected leaves in previous autumn [4]. In this context, the application of crystalline urea has been shown to improve leaf decomposition, reducing the overwintering inoculum [18]. However, it is also important to know the meteorological conditions affecting ascospore formation and release, to identify the best period for the treatment of the leaf litter [19]. Chemical control is instead based on the application of fungicides from petals fall until the end of summer [3,20,21]. However, the application of chemical means is recently limited by the restricted availability of authorized fungicides to enable sustainable crop protection in agriculture [22]. Then, the adoption of tolerant cultivars, in combination with the agronomic practices focused on the reduction of the primary inoculum, represents an important strategy for RLB management, reducing the application of chemical means [14,23].

The objectives of this study were to expand current knowledge on RLB in Apulia by investigating: (i) the susceptibility of 31 local almond cultivars, compared with commercial cultivars (‘Belona’, ‘Genco’, ‘Guara’—marketed as ‘Tuono’ in Italy [24], ‘Ferragnès’, ‘Filippo Ceo’, ‘Lauranne^®^ Avijor’, ‘Soleta’, and ‘Supernova’); (ii) the identity of the causal agent responsible for RLB in Apulian orchards; and (iii) the environmental conditions promoting RLB recrudescence in Apulian almond-growing areas.

## 2. Results

### 2.1. Susceptibility of Almond Cultivars to Red Leaf Blotch

Monitoring in Field 1 were conducted over the 2023–2025 growing seasons. Based on symptom development across all almond cultivars, the first RLB symptoms in 2023 were observed between the third decade of March and the first decade of April. In contrast, in 2024 and 2025, the first symptoms appeared later, between the third decade of May and the first decade of June.

In 2023 and 2025, symptoms caused by *P. amygdalinum* were generally scarce and limited to a few cultivars. In 2023, symptoms were detected only on ‘Rachele piccola’ (5.5%) and ‘Sammichelana’ (2.4%). In 2025, infections were recorded on ‘Della Madonna di Molfetta’ (1%), ‘Carolina Tribuzio’ (0.75%), ‘Canasce’ (0.1%), and ‘Sammichelana’ (0.1%). Symptom severity never exceeded class 2 in 2023 (for ‘Rachele piccola’ and ‘Sammichelana’) and class 1 in 2025 (for ‘Della Madonna di Molfetta’, ‘Carolina Tribuzio’, ‘Canasce’, and ‘Sammichelana’).

**Figure 1:**
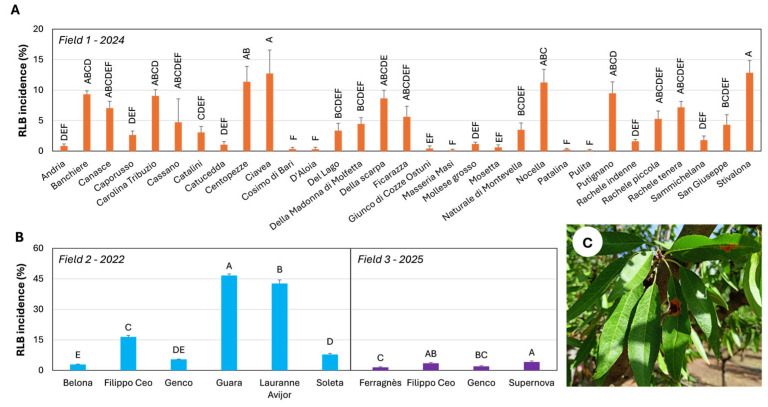
Incidence of Red Leaf Blotch (RLB) in almond cultivars evaluated in Field 1 (**A**) and in commercial orchards (Fields 2 and 3) (**B**), with representative symptoms on leaves (**C**). (**A**) RLB incidence assessed in Field 1 recorded in 2024. (**B**) RLB incidence in Field 2 during the 2022 growing season, and in Field 3 during the 2025 growing season. Bars indicate mean ± SE (n = 8 in Field 1; n = 12 in Fields 2 and 3). The same letters, indicate in the same field, mean values statistically no differentiable according to Tukey’s HSD test at the probability level of 0.01.

In 2024, RLB symptoms were more prevalent, enabling statistical comparisons among cultivars. ‘Stivalona’ and ‘Ciavea’ showed the highest incidence (12.8% and 12.7%, respectively), differing significantly from all other cultivars. ‘Centopezze’, ‘Nocella’, ‘Putignano’, ‘Banchiere’, ‘Carolina Tribuzio’, ‘Della Scarpa’, ‘Rachele tenera’, and ‘Canasce’ showed incidences between 11.4% and 7.1%. Conversely, ‘Catucedda’, ‘Cosimo di Bari’, ‘D’Aloia’, ‘Giunco di Cozze’, ‘Ostuni’, ‘Masseria Masi’, ‘Mollese grosso’, ‘Mosetta’, ‘Patalina’, and ‘Pulita’ exhibited incidences below 2%. Intermediate responses were recorded in the remaining cultivars (Figure 1A).

Surveys in commercial orchards were conducted in 2022 (Field 2) and 2025 (Field 3). In Field 2, ‘Guara’ and ‘Lauranne^®^ Avijor’ were the most susceptible cultivars, with mean incidences of 46.7% and 42.7%, respectively. ‘Filippo Ceo’ displayed an intermediate response, with incidences not exceeding 16.5%, whereas ‘Belona’, ‘Genco’, and ‘Soleta’ were the most tolerant, exhibiting incidences between 5.4% and 2.9%.

In Field 3, RLB symptoms were generally moderate, with incidence never exceeding 5%. Nonetheless, significant differences among cultivars were detected (*p* < 0.01). ‘Supernova’ and ‘Filippo Ceo’ exhibited incidences of 4.1% and 3.5%, respectively. ‘Genco’ showed lower incidence (2.0%), followed by ‘Ferragnès’ (1.5%), which ranked as the most tolerant cultivar (Figure 1B).

Regarding symptom severity in Field 1 during 2024, no cultivar exhibited an average severity score higher than class 3. The highest mean severity values were recorded for ‘Centopezze’ (2.6), ‘Ciavea’ (2.5), and ‘Stivalona’ (2.3). Pearson’s correlation analysis revealed a strong, positive, and statistically significant relationship between disease incidence and symptom severity (r = 0.83). The test statistic (t = 8.15, df = 29) yielded a highly significant *p*-value (*p* ≈ 5.4 × 10^−9^), and the 95% confidence interval (0.68–0.92) confirmed the robustness of this association (Figure 1A).

To further characterize cultivar behaviour in Field 1, a cluster analysis was conducted using the I and Se values from the 2024 survey. The NbClust function identified two distinct groups. As shown in Figure S1B, ten cultivars clustered as susceptible, with mean incidence (I) and severity (Se) values of 9.9% and 2.1, respectively, while twenty-one cultivars grouped as tolerant, with markedly lower mean incidence (0.2%) and severity (0.9).

The boxplot in Figure S1C depicts the distribution of infected leaf percentages according to cultivar flowering time in Field 1 (2024). Although differences among flowering groups were not statistically significant (Kruskal–Wallis: χ^2^ = 1.92; *p* = 0.59), a slight tendency was observed: early- and intermediate-flowering cultivars exhibited marginally higher mean and median incidence values, whereas late-flowering cultivars generally showed the lowest infection levels.

### 2.2. Relationships Between Red Leaf Blotch and Meteorological Conditions

Meteorological data are summarized in Figure S2. The months during which field surveys were conducted are highlighted using coloured rectangles to indicate the onset and intensity of symptoms: orange denotes periods with low disease incidence and frequency, whereas red indicates months characterized by higher incidence and greater symptom severity.

**Figure 2:**
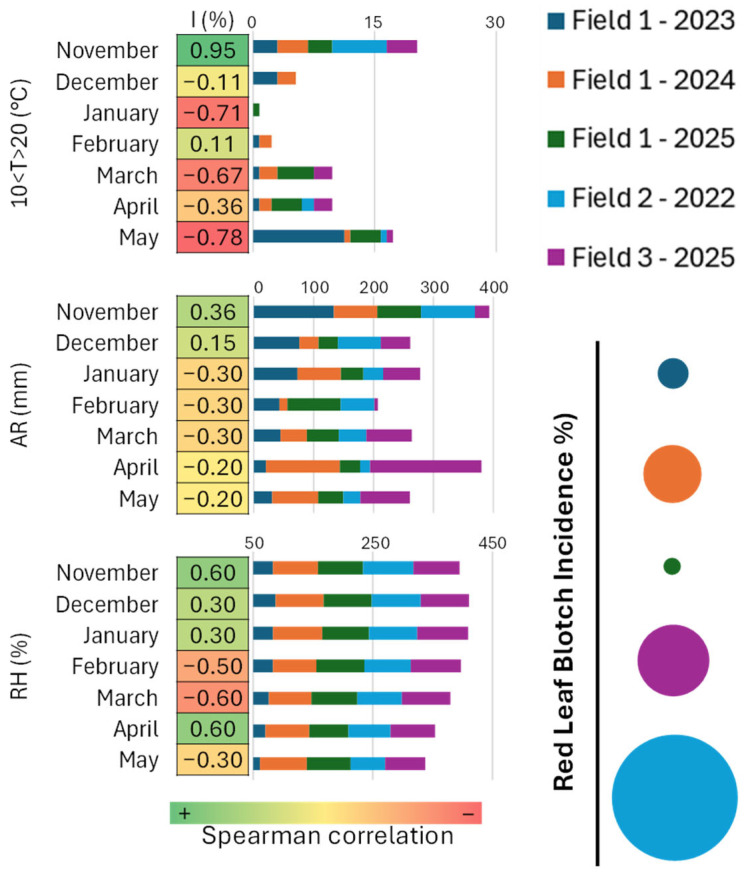
Spearman’s correlation between meteorological variables and I (percentage of infected leaves). Meteorological variables considered for each Field include: number of days with temperatures between 10 °C and 20 °C, accumulated rainfall (AR), and mean relative humidity (RH) for each month from November to May. In the bar chart depicting the meteorological data and in the circle chart summarizing the average percentage of infected leaves, colours indicate the Field and corresponding monitoring year. For Field 1, data for 2023, 2024, and 2025 are represented in blue, orange, and green, respectively; Field 2 (2022) is shown in light blue; Field 3 (2025) is shown in purple. Circle size is proportional to the average percentage of infected leaves recorded in each Field and year.

Spearman’s correlation was applied to explore the relationship between meteorological variables and disease incidence, taking into consideration the three Fields under study and all the growing seasons. The heatmap in Figure 2 shows the different Spearman coefficients for each meteorological variable considered. It is interesting to note that temperatures in the range 10–20 °C, average relative humidity and accumulated rainfall in November preceding the onset of symptoms, together with relative humidity in April, have a positive correlation with the percentage of infected leaves (I) (0.95, 0.36, 0.60, 0.60, respectively).

### 2.3. Morphological and Molecular Characterization of Polystigma amygdalinum

**Figure 3:**
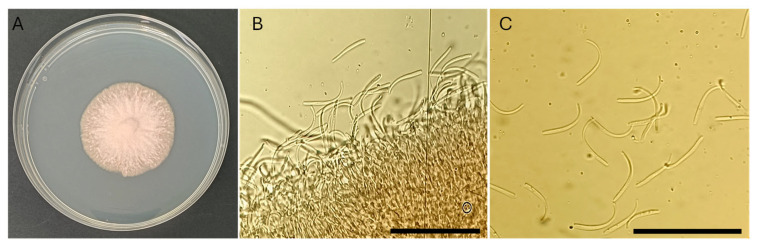
*Polystigma amygdalinum* isolated from almond leaves. (**A**) Twenty-day-old colony grown on PDA at 24 °C. (**B**) Stromatic tissue observed at 400× magnification. (**C**) Conidia at 400× magnification. Scale bars: (**B**,**C**) = 100 µm. Images were acquired using an Olympus BH2 BHS-312 trinocular microscope.

Due to the hemibiotrophic nature of the fungus, isolation on agar media was particularly challenging, yielding only small and slow-growing colonies. From leaves exhibiting early RLB symptoms, a colony of approximately 4 cm in diameter developed after ~20 days of incubation at 25 °C (Figure 3A). The culture produced stromata, hyphae, and conidia consistent with the diagnostic characteristics of *P. amygdalinum* (Figure 3B,C). Distinct septa were not clearly visible in all hyphae. Conidia were filiform, hyaline, aseptate, and slightly curved at one end, with an average length ranging from 37.56 to 42.18 µm and an average width between 1.54 and 1.62 µm (Figure 3C). Conidia were observed in greatest abundance near the conidiogenous cells (Figure 3B).

An amplicon of the expected size (560 bp) was obtained by PCR amplification of DNA extracted from pure mycelium of isolates GPL1 and GPL2 of *P. amygdalinum*, using the primer pair PyITS1/ITS4. BLASTn (Basic Local Alignment Search Tool, BLAST+ version 2.17.0) analysis showed that isolate GPL1 exhibited 100% identity (100% coverage) with *P. amygdalinum* isolate TO10 (accession MH205939) and 99.77% identity (100% coverage) with several other isolates, including KARE2768 (PV491258), KARE2773 (PV491260), KARE2771 (PV491259), KARE2767 (PV491257), and M4 (KC756362). Isolate GPL2 also displayed 100% identity (100% coverage) with multiple *P. amygdalinum* isolates (e.g., TO1 MH205935; KARE2768 PV491258; KARE2773 PV491260; KARE2771 PV491259; KARE2767 PV491257; M4 KC756362) and 99.77% identity (100% coverage) with isolates Y1 (KC756366), MM1 (KC756365), TO10 (MH205939), HB3-550 (JQ995323), and EA1 (KC756360).

Amplification of DNA from symptomatic leaf samples (Leaf3 and Leaf38), using the *P. amygdalinum*-specific primer pair PamyI2F4/I2R2, yielded the expected 99 bp fragment. Both sequences showed 100% identity (89% coverage) with multiple *P. amygdalinum* isolates, including KARE2768 (PV491258), MM1 (KC756365), TO10 (MH205939), and Y1 (KC756366).

**Figure 4:**
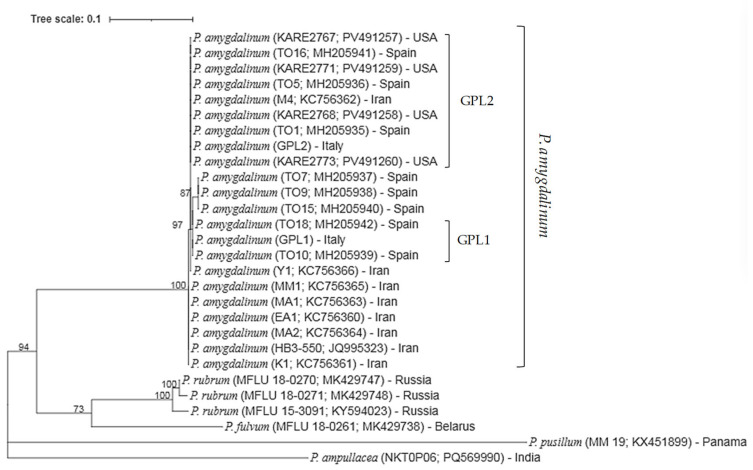
Maximum Likelihood phylogenetic tree depicting the relationships among *Polystigma* species, including *P. amygdalinum* isolates collected from infected almond leaves in southern Italy, based on partial ITS region sequences. The analysis was performed using the Tamura–Nei substitution model. Bootstrap support values (>70%) from 1000 replicates are shown at the corresponding nodes. The scale bar represents 0.10 nucleotide substitutions per site.

Phylogenetic analysis was conducted using the ITS sequences obtained from pure cultures of *P. amygdalinum*. All reference and newly generated sequences clustered within a single, well-supported clade, with a bootstrap value of 100%. Isolate GPL1 (obtained from cv. ‘Guara’, exhibiting early RLB symptoms) grouped with reference isolates TO10 and TO18 with 97% bootstrap support, whereas isolate GPL2 (from cv. ‘Lauranne^®^ Avijor’) clustered with reference isolates TO1, KARE2768, and M4, supported by an 87% bootstrap value. These results confirm the identity of the isolates as *P. amygdalinum* (Figure 4).

### 2.4. Detection of Polystigma amygdalinum on Almond Leaves by qPCR

**Figure 5:**
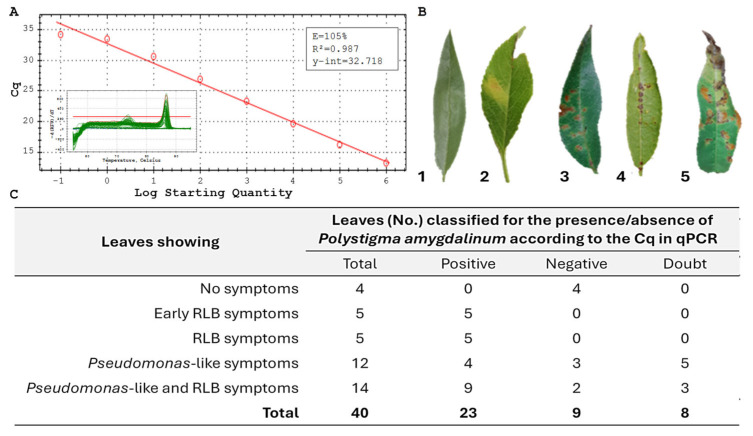
(**A**) Standard curve generated using *Polystigma amygdalinum* DNA serial decimal dilutions from 20 ng µL^−1^ to 2 fg µL^−1^. The inset shows the melt curve profile for the 40 analysed almond leaf samples, confirming the presence of a single, specific amplification peak. (**B**) Representative symptoms observed on almond leaves: 1 = asymptomatic (healthy) leaf; 2 = early Red Leaf Blotch (RLB) symptoms; 3 = fully developed RLB symptoms; 4 = *Pseudomonas*-like symptoms; 5 = mixed symptoms (*Pseudomonas*-like + early RLB). (**C**) Detection of *Polystigma amygdalinum* in almond leaves with different symptom types using qPCR with the specific primer pair PamyI2F4/PamyI2R2. Samples with Cq ≤ 30 were classified as positive, those with Cq ≥ 35 as negative, and samples with 30 < Cq < 35 as doubtful. The positive control exhibited a Cq of 17.06, while the negative control showed no amplification (NA).

Based on the standard curve generated using DNA from the reference strain, Cq threshold values were established to discriminate positive, doubtful, and negative samples for the presence of *P. amygdalinum* DNA, following a diagnostic qPCR approach. Using DNA from *P. amygdalinum* (syn. *Polystigma ochraceum*, CBS 320.58) within the concentration range of 20 to 2 × 10^−6^ ng µL^−1^, threshold Cq values were established (Figure 5A). Samples with Cq ≤ 30 were considered positive, those with Cq ≥ 35 were classified as negative, while samples with Cq values between 30 and 35 were regarded as doubtful. As shown in the inset of Figure 5A, the melt curve analysis confirmed the presence of a single, specific melting peak, indicating the absence of non-specific amplification.

A total of 40 almond leaves exhibiting different symptom categories—(i) well-defined RLB symptoms, (ii) early RLB symptoms, (iii) *Pseudomonas*-like symptoms, (iv) mixed *Pseudomonas*-like and early RLB symptoms, and (v) asymptomatic leaves (Figure 3B)—were assessed by qPCR following the protocol of [22]. The results are summarized in Figure 5C. *P. amygdalinum* was detected in 100% of samples showing clear RLB symptoms as well as those with early RLB symptoms. The pathogen was also detected in 4 out of 12 samples exhibiting Pseudomonas-like symptoms and in 9 out of 14 samples with mixed *Pseudomonas*-like and early RLB symptoms.

## 3. Discussion

In recent years, *Polystigma amygdalinum* has become an increasingly significant problem across the Mediterranean Basin and the Middle East. Multiple studies have focused on elucidating the pathogen’s life cycle and cultivar-specific responses to infection, with the ultimate goal of improving disease management. Within this context, the availability of tolerant cultivars is strategically important in regions where climatic conditions strongly favour Red Leaf Blotch (RLB) epidemics. Several widely cultivated almond cultivars—such as ‘Belona’, ‘Ferragnès’, ‘Genco’, ‘Guara’, ‘Lauranne^®^ Avijor’, ‘Soleta’, and ‘Supernova’—have been evaluated for their susceptibility to RLB.

In our study, the cultivar ‘Guara’/’Tuono’, an intermediate-flowering genotype, consistently emerged as highly susceptible, in agreement with earlier reports by [11,14,25]. In contrast, ‘Belona’, ‘Ferragnès’, and ‘Supernova’—intermediate and late-flowering cultivars—consistently displayed high tolerance, aligning with previous studies and confirming their stability across diverse environments [11,14]. Notably, our findings diverged from Miarnau et al. (2021) [14] regarding ‘Lauranne^®^ Avijor’, which in our trials showed susceptibility comparable to ‘Guara’/’Tuono’. Similarly, ‘Soleta’, previously characterised as susceptible, exhibited only moderate susceptibility in our study. Remarkably, Genco’, a medium-late flowering cultivar, showed strong tolerance, contrary to the classification by Ollero-Lara et al. (2019) [11], who reported it as susceptible. These discrepancies raise relevant questions about whether certain genotypes may display different RLB phenotypes depending on climatic zone or local environmental conditions. To our knowledge, this is also the first study demonstrating that ‘Filippo Ceo’ (intermediate-flowering cultivar) is tolerant to RLB.

In Field 1, cultivars representative of Apulian almond biodiversity were evaluated to identify genotypes potentially tolerant to *P. amygdalinum*. A noteworthy outcome was the strong association between flowering time and RLB incidence. Higher disease incidence was predominantly observed in cultivars flowering in mid-February, whereas late and medium-late flowering cultivars (flowering from late February to early March) showed significantly greater tolerance. This pattern is consistent with the findings of Gort (2014) [15] and Ollero-Lara et al. (2019) [11] and emphasizes the importance of phenology in disease management guidelines. A plausible explanation is that early-flowering cultivars coincide with the peak release of mature ascospores and experience a longer susceptibility window than later-flowering genotypes.

The three-year survey in Field 1 revealed that RLB pressure was greatest in 2024. During this season, a substantial cluster of cultivars—including ‘Andria’, ‘Mosetta’, ‘Catucedda’, ‘Mollese Grossa’, ‘Rachele Indenne’, ‘Sammichelana’, ‘Cosimo di Bari’, ‘D’Aloia’, ‘Patalina’, ‘Giunco di Cozze’, ‘Masseria Masi’, and ‘Pulita’—grouped as highly tolerant, with a mean incidence of only 0.73% infected leaves. Importantly, all these cultivars are characterised by late or medium-late flowering, further reinforcing the phenological pattern observed.

Analyses of meteorological variables associated with RLB development revealed consistent associations between disease incidence and specific thermo-hygrometric conditions occurring in autumn, winter, and spring. However, given the exploratory nature of the correlation analysis and the inherent collinearity among meteorological variables, these results should be interpreted as indicative of favourable climatic patterns rather than as evidence of independent or causal effects. Mild temperatures and reduced humidity—such as those recorded in Corato in 2022 and Locorotondo in 2024—favoured higher disease pressure, whereas cooler and wetter conditions in Locorotondo during 2023 and 2025 were associated with minimal disease incidence. According to [1,8], mild temperatures at leaf fall and adequate humidity are associated with perithecia maturation, while spring temperature–precipitation interactions are associated with ascospore release. Our observations are consistent with these epidemiological frameworks, although the present study does not allow formal testing of the independent contribution of each climatic variable. Previous studies [2,8,26] have also shown that in spring, high humidity—particularly following rainfall—facilitates the discharge of mature ascospores capable of infecting new leaves. Research from Iran [27] indicated that winter temperatures below 10 °C are important for perithecia maturation, while more recent studies in Spain [13] demonstrated that meteorological conditions from October to January strongly influence the total ascospore load in the subsequent season. These authors reported a positive correlation with relative humidity, cumulative rainfall, the number of rainy days, and the number of days above 20 °C. Our results indicate a positive association between RLB incidence and specific thermo-hygrometric conditions, particularly those occurring in November preceding symptom expression, including accumulated rainfall, mean relative humidity, and the number of days with temperatures between 10 and 20 °C, as well as relative humidity in April. In Ruvo di Puglia, Corato, and Locorotondo during the 2024 season, these climatic conditions co-occurred with higher disease levels. These associations highlight potentially favourable environmental windows for RLB development, rather than identifying independent or causal drivers [27,28].

With regard to molecular detection of *P. amygdalinum* in leaves displaying early or ambiguous symptoms, the applied qPCR protocol successfully detected the pathogen in early RLB stages and in leaves exhibiting mixed infections or *Pseudomonas*-like symptoms. More than 33% of leaves showing solely *Pseudomonas*-like symptoms tested positive for *P. amygdalinum*, with the percentage rising to 64% in samples exhibiting mixed symptoms. These results indicate the presence of *P. amygdalinum* DNA in leaves with atypical symptomatology, but do not demonstrate a causal role of the fungus in symptom development in the absence of isolation or pathogenicity tests. This finding highlights the difficulty of distinguishing overlapping foliar disorders in the field and underscores the diagnostic value of molecular tools for detecting early or latent infections.

Overall, this research provides novel insights into RLB epidemiology in the Apulia region and on the behaviour of the widely cultivated ‘Filippo Ceo’, as well as on the susceptibility of 31 almond cultivars representative of local Apulian germplasm. The three-year dataset demonstrates that a substantial number of local cultivars show greater tolerance than the globally widespread ‘Guara’/‘Tuono’, representing a valuable genetic reservoir for improving the resilience of commercial cultivars in the context of sustainable agriculture. Furthermore, this study confirms that RLB susceptibility is strongly flowering-time dependent, with early-flowering cultivars notably more susceptible than medium-late and late-flowering ones. These findings will be of considerable value for forecasting model development and practical disease management. Finally, *P. amygdalinum* was confirmed as the only fungal species molecularly associated with leaves exhibiting typical RLB symptoms in Apulia. However, its frequent molecular detection in leaves showing non-typical or *Pseudomonas*-like symptoms suggests unresolved epidemiological questions, including possible latent colonization or co-occurrence with other biotic agents, which warrant further investigation.

In conclusion, to address the main objectives of this study, the key findings are summarised as follows: (i) in the seasons particularly favorable to the disease development, the commercial cultivars ‘Belona’, ‘Genco’ and ‘Soleta’ and the local cultivars ‘Cosimo di Bari’, ‘D’Aloia’, ‘Masseria Masi’, ‘Patalina’ and ‘Pulita’ were the most tolerant to the RLB; (ii) as reported in previous studies [8,9,19], autumn–spring thermo-hygrometric conditions were consistently associated with variations in epidemic intensity, supporting their consideration in forecasting and management strategies. However, further studies integrating multivariate and modelling approaches will be necessary to separate the relative contribution of individual climatic variables and their interaction with site- and cultivar-specific factors; (iii) the qPCR assay developed by [29] proved to be a reliable diagnostic tool for the detection of *P. amygdalinum* in almond leaves, also useful for identifying early or atypical infections, without implying quantitative assessment of pathogen load or gene expression.

Finally, *P. amygdalinum* was confirmed as the sole fungal agent consistently associated with typical RLB symptoms in Apulian almond orchards. Nevertheless, its detection in leaves exhibiting atypical or mixed symptomatology indicates that additional biotic factors or early asymptomatic colonization may occur, highlighting the need for further targeted epidemiological and pathogenicity studies.

## 4. Materials and Methods

### 4.1. Almond Fields and Sampling

This study was conducted on thirty-nine almond cultivars (Table 1) grown in an ex situ biodiversity germplasm collection (Field 1) located in Locorotondo (BA, Apulia, Italy; 40°45′27.2″ N, 17°20′22.3″ E) and in two commercial orchards (Fields 2 and 3) situated in Corato (BA, Apulia, Italy; 41°03′45.7″ N, 16°18′06.5″ E) and Ruvo di Puglia (BA, Apulia, Italy; 41°03′02.9″ N, 16°28′55.2″ E), respectively.

In the ex situ collection (Field 1), each cultivar is represented by two trees planted in 2017 at a spacing of 4 × 4 m and managed under rainfed conditions following standard regional cultural practices. Soil management, pruning, and fertilization were carried out according to the Phytosanitary Regulations of the Apulia Region, and no fungicides were applied during the study period (2023–2025).

In the commercial orchards, the six cultivars in Field 2 and the four cultivars in Field 3 were planted in 2005 and 2007, respectively, at 5 × 5 m spacing. Both orchards are managed according to organic farming principles in compliance with European Regulation (EU) No. 2018/848 [30]. Copper-based fungicides were routinely applied to manage foliar and fruit diseases. For RLB assessment and sampling in Fields 2 and 3, a randomized block design was implemented, consisting of four blocks per cultivar, each with twelve replicated trees.

### 4.2. Disease Assessment

RLB symptoms were assessed from late March to late August on four branches per plant. For each branch, 100 leaves were individually examined, and these observations were used to calculate disease incidence (I; percentage of infected leaves) for each plant. Disease severity (Se) was evaluated on 25 fully expanded leaves per branch using the empirical scale proposed by Miarnau et al. (2021) [14], which includes five classes: class 0 = no symptoms; class 1 = 1–10% infected leaf area i.l.a.; class 2 = 11–20% i.l.a.; class 3 = 21–50% i.l.a.; class 4 ≥ 0% i.l.a.

### 4.3. Meteorological Data

Meteorological data for Field 1 were obtained from an automatic weather station (Neetra IoT Agrismart ID Centralina C6C240, 40°45′24.5″ N, 17°20′27.7″ E) connected to the Netsens monitoring system [31]. For Fields 2 and 3, meteorological variables were retrieved from NASA’s Prediction Of Worldwide Energy Resources database [32]. Recorded variables included monthly minimum, maximum, and mean temperatures, total monthly precipitation (mm), and mean monthly relative humidity (%). From these data, the monthly number of days with temperatures between 10 and 20 °C and cumulative rainfall were derived.

### 4.4. Isolation of Polystigma amygdalinum and Growth Conditions

Approximately twenty leaves showing early and well-developed RLB symptoms were selected for fungal isolation. Leaves were surface-sterilized by immersion in 70% ethanol for 30 s, rinsed twice in sterile distilled water and air-dried under aseptic conditions. Small tissue sections excised from the marginal of symptomatic areas were placed on Potato Dextrose Agar (PDA; Difco™ Becton Dickinson, East Rutherford, NJ, USA) supplemented with streptomycin sulphate (500 mg/L; Sigma-Aldrich Merck Life Science S.r.l., Milan, Italy). After 20 days of incubation at 25 °C in the dark, a few pink-coloured fungal colonies were transferred to fresh PDA plates.

Hyphae and conidia were mounted in sterile water and examined using an optical microscope (Olympus BH2 BHS-312 trinocular microscope; Olympus Europa SE & Co. KG, Hamburg, Germany). Morphological features of the isolates were compared with those of the reference strain *P. amygdalinum* (syn. *P. ochraceum*) CBS 320.58.

### 4.5. DNA Extraction, PCR, and qPCR Assays

Leaves displaying: (i) unequivocal RLB symptoms, (ii) ambiguous RLB symptoms, (iii) symptoms consistent with *Pseudomonas* infection, or (iv) mixed *Pseudomonas*-like and early RLB symptoms, were collected in Field 1. A total of forty symptomatic leaves, independent of cultivar, were analysed.

DNA was extracted according to [33] with minor modifications. In brief, 0.5 g of leaf tissue was placed in an extraction bag (12 × 15 cm; Bioreba AG, Reinach, Switzerland) with 5 mL of CTAB extraction buffer and homogenised using a HOMEX 7 system (Bioreba AG). One millilitre of the macerate was transferred to a 2 mL microtube and incubated at 65 °C for 30 min. DNA was extracted through two consecutive chloroform washes and centrifugation at 14,000 rpm at 4 °C. The supernatant was transferred to a new tube, mixed with 0.7 volumes of cold isopropanol, and incubated at −20 °C for 30 min. Following centrifugation (14,000 rpm, 25 min, 4 °C), the pellet was washed with 70% ethanol, centrifuged again (14,000 rpm, 5 min, 4 °C), vacuum-dried, and resuspended in 50 µL of nuclease-free water.

Genomic DNA from the three putative *P. amygdalinum* isolates and from the reference strain CBS 320.58 was extracted from 20-day-old cultures grown in Potato Broth at 25 °C with shaking at 200 rpm. Mycelium was collected by filtration through sterile filter paper, washed twice in sterile distilled water, and processed following [34].

DNA quantity and quality were measured using a NanoDrop 2000 spectrophotometer (Thermo Fisher Scientific Inc., Wilmington, DE, USA) and a Qubit 2.0 fluorometer (Life Technologies Ltd., Paisley, UK) with the dsDNA BR Assay Kit (Thermo Fisher Scientific Inc.). All DNA samples were stored at −20 °C until use.

Primer pairs used for end-point PCR and qPCR assays, targeting the ITS region, are listed in Table S1.

The identity of the fungus was initially verified by end-point PCR. Each reaction (25 μL total volume) contained 12.5 μL of Taq PCR 2× Master Mix (Qiagen, Milan, Italy), 1.5 μL of MgCl_2_ (25 mM), 0.2 μL of each primer (25 µM), 2 μL of template DNA (25 ng/μL), and ultrapure water to volume. PCR amplification was performed using a Bio-Rad T100 Thermal Cycler (Bio-Rad Laboratories, Hercules, CA, USA) following the cycling conditions described by [4].

The qPCR assay was applied exclusively for diagnostic detection of *P. amygdalinum* DNA in almond leaf tissues. Amplification results were interpreted based on Cq threshold values and melting curve specificity, without the use of reference genes, calibrator samples, or relative quantification methods. The primer pair PamyI2F4/PamyI2R2 was employed to distinguish early RLB symptoms from those caused by other pathogens such as *Pseudomonas* spp. Each 25 μL reaction consisted of 10 μL of SsoAdvanced™ Universal SYBR^®^ Green Supermix (Bio-Rad Laboratories), 0.08 μL of each primer (25 µM), 2 μL of DNA (25 ng/μL), and ultrapure water to volume. Amplification was performed on a CFX Connect Real-Time PCR System (Bio-Rad Laboratories) according to Zúñiga et al. (2018) [29], with the following modifications: primer concentration was reduced to 80 nM, annealing temperature increased to 62 °C, and the number of cycles reduced to 36.

All reactions were performed in triplicate. A no-template control (NTC; ultrapure sterile water) and a positive template control (PTC; *P. amygdalinum* (*P. ochraceum*) CBS 320.58 DNA, 25 ng/μL) were included in each run. A melting curve analysis from 55 °C to 95 °C, with 0.1 °C increments, was carried out at the end of each qPCR assay to verify product specificity and detect possible non-specific amplification.

### 4.6. Sequencing and Phylogenetic Analyses

PCR amplicons from two representative isolates morphologically identified as *P. amygdalinum* and from two leaves exhibiting clear and well-developed RLB symptoms were sequenced in both forward (5′→3′) and reverse (3′→5′) directions by an external sequencing service (Macrogen, Seoul, Republic of Korea). Consensus sequences were assembled using BioEdit Sequence Alignment Editor (BioEdit v7.7.1.0; last update 5 October 2021, Tom Hall) and subsequently identified through BLASTn searches against the NCBI nucleotide database [35].

Multiple sequence alignment of the partial ITS region was conducted using the sequences generated in this study (*P. amygdalinum* isolates GPL1 and GPL2), reference sequences of *P. amygdalinum* and other *Polystigma* spp., and *Pseudopestalotiopsis ampullacea* as outgroup, all retrieved from GenBank (Table S2). Alignments were produced with the CLUSTALW algorithm [36] implemented in MEGA v12 [37].

Phylogenetic reconstruction was performed using the Maximum Likelihood (ML) method based on the Tamura–Nei model of nucleotide substitution [38]. Branch support was assessed using 1000 bootstrap replicates, and bootstrap values are indicated next to the corresponding branches [39]. For the heuristic search, the initial tree was selected based on the highest log-likelihood score among those obtained using the Neighbor-Joining (NJ) method [40] and the Maximum Parsimony (MP) approach.

### 4.7. Data Analysis

The mean incidence (I) of RLB and the corresponding standard errors for the different almond cultivars were represented using histograms in Figure 1. Significant differences among cultivars were assessed separately for each field by applying one-way analysis of variance (ANOVA), followed by Tukey’s Honestly Significant Difference (HSD) test at the significance level (0.01) selected based on ANOVA outcomes. In Field 1, where surveys were carried out over three consecutive years (2023, 2024, and 2025), cultivar comparisons were performed independently for each year. All analyses, including tests of normality, were conducted using JMP^®^ Pro (version 18; SAS Institute Inc., Cary, NC, USA, 1989–2023).

In Field 1, Pearson’s correlation analysis was used to investigate the relationship between incidence (I) and severity (Se). The correlation plot was generated in RStudio (version 2024.12.1 [41]) using the ggplot2 package. Based on the same dataset, cultivar tolerance to RLB was evaluated using the NbClust and k-means algorithms in RStudio to determine and visualise the optimal grouping of cultivars, with graphical outputs produced using the factoextra package.

Additionally, in Field 1, incidence data were grouped according to cultivar flowering period and visualised using boxplots generated with ggplot2. Data distribution within flowering groups was first evaluated using the Shapiro–Wilk normality test. As normality assumptions were not met, differences among groups were assessed using the Kruskal–Wallis test, followed by Dunn’s post hoc test with Bonferroni correction, implemented via the FSA package in RStudio.

Finally, RLB incidence data from all fields (2022–2025) were analysed in relation to meteorological variables using Spearman’s rank correlation, computed with the cor() function in RStudio. This non-parametric approach was applied as an exploratory tool to identify temporal associations between disease incidence and climatic conditions, without attempting to separate the independent effects of collinear meteorological variables or site- and year-specific factors.

## Figures and Tables

**Table 1:** Origin and main characteristics of the almond cultivars under study.

ID *	Cultivar	Origin	Flowering	Ripening (Decade)	Field **
1	Andria	Barletta_Andria_Trani	Late	2nd of September	1
2	Banchiere	Monte Sant’Angelo	Medium-late	2nd of September	1
3	Canasce	Andria	Early	3rd of August	1
4	Caporusso	Acquaviva delle Fonti	Intermediate	2nd of September	1
5	Carolina Tribuzio	Acquaviva delle Fonti	Intermediate	1st of September	1
6	Cassano	Cassano delle Murge	Medium-late	1st of September	1
7	Catalini	Bitetto	Late	1st–2nd of September	1
8	Catucedda	Alberobello	Intermediate	1st of September	1
9	Centopezze	Monopoli	Early	1st of September	1
10	Ciavea	Massafra	Early	3rd of August	1
11	Cosimo di Bari	Toritto	Late	2nd of September	1
12	D’Aloia	Valenzano	Intermediate	3rd of August	1
13	Del Lago	Molfetta	Intermediate	1st of September	1
14	Della Madonna di Molfetta	Molfetta	Medium-late	1st of September	1
15	Della Scarpa	Conversano	Intermediate	1st of September	1
16	Ficarazza	Ruvo di Puglia	Early	1st of September	1
17	Giunco di Cozze Ostuni	Ostuni	Early	1st of September	1
18	Masseria Masi	Putignano	Early	1st of September	1
19	Mollese Grosso	Ceglie Messapica	Medium-late	1st–2nd of September	1
20	Mosetta	Trani	Intermediate	2nd of September	1
21	Naturale di Montevella	Acquaviva delle Fonti	Medium-late	2nd of September	1
22	Nocella	Ruvo di Puglia	Intermediate	1st of September	1
23	Patalina	Putignano	Late	2nd–3rd of August	1
24	Pulita	Trani	Medium-late	1st–2nd of September	1
25	Putignano	Molfetta	Early	3rd of August	1
26	Rachele Indenne	Bari	Early	1st of September	1
27	Rachele Piccola	Triggiano	Early	1st of September	1
28	Rachele Tenera	Crispiano	Intermediate	1st of September	1
29	Sammichelana	Sammichele di Bari	Late	1st of September	1
30	San Giuseppe	Ceglie Messapica	Early	2nd–3rd of August	1
31	Stivalona	Castellana Grotte	Late	1st of September	1
32	Belona	Spanish	Intermediate	2nd–3rd of August	2
33	Ferragnès	French	Late	2nd of September	3
34	Filippo Ceo	Apulia	Intermediate	1st of September	2,3
35	Genco	Taranto	Medium-late	2nd of September	2
36	Guara	Spanish	Intermediate	3rd of August	2,3
37	Lauranne^®^ Avijor	French	Late	1st of September	2
38	Soleta	Spanish	Intermediate	3rd of September	2
39	Supernova	Rome	Late	1st of September	3

* ID: cultivar code number; ** Field 1: ex situ biodiversity germplasm collection experimental field (Centro di Ricerca, Sperimentazione e Formazione “Basile Caramia”, Locorotondo BA, Apulia, Italy, 40°45′27.2″ N 17°20′22.3” E); Field 2: commercial orchard (Corato, BA, Apulia, Italy, 41°03′45.7″ N 16°18′06.5″ E); Field 3: commercial orchard (Ruvo di Puglia, BA, Apulia, Italy, 41°03′02.9″ N 16°28′55.2″ E).

## Data Availability

The nucleotide sequences of *Polystigma amygdalinum* generated in this study are not publicly available and were not deposited in a public repository. All other data are available within the article.

## Supplementary Materials

The following supporting information can be downloaded at: https://www.mdpi.com/article/10.3390/plants15020188/s1, Figure S1: (A) Correlation between incidence (I) and severity (Se) of Red Leaf Blotch (RLB). Each point represents the average incidence and severity values for one of the 31 almond cultivars grown in Field 1. (B) Cluster analysis of almond cultivars based on I and Se values recorded in 2024. The optimal number of clusters was determined using the NbClust algorithm, and clustering was performed using the k-means method. Cluster 1 (green) corresponds to less susceptible cultivars, whereas Cluster 2 (red) comprises more susceptible cultivars. Cultivar identification numbers correspond to those listed in Table 1. (C) Percentage of infected leaves across flowering-period categories (early, intermediate, medium–late, late). Each boxplot shows the distribution of infection levels within each flowering group; the horizontal line indicates the median, and the “X” denotes the mean; Figure S2: (A–C) Meteorological data recorded in Field 1 (ex situ biodiversity germplasm collection experimental field at the Centro di Ricerca, Sperimentazione e Formazione in Agricoltura “Basile Caramia”, Locorotondo, BA, Apulia, Italy; 40°45′27.2″ N, 17°20′22.3″ E) for the years 2023, 2024, and 2025, obtained from the local automatic meteorological station (Neetra IoT Agrismart, ID Centralina C6C240, 40°45′24.5″ N, 17°20′27.7″ E). (D) Meteorological data for Field 2 (commercial orchard, Corato, BA, Apulia, Italy; 41°03′45.7″ N, 16°18′06.5″ E) for 2021–2022. (E) Meteorological data for Field 3 (commercial orchard, Ruvo di Puglia, BA, Apulia, Italy; 41°03′02.9″ N, 16°28′55.2″ E) for 2024–2025. For Fields 2 and 3, meteorological data were retrieved from the NASA Prediction of Worldwide Energy Resources database [33]. (F) Map of the Apulia region showing the geographical location of the three study sites; Table S1: Details on the primer sets used; Table S2: GenBank reference sequences used for the phylogenetic analysis of *Polystigma* spp. and the outgroup *Pseudopestalotiopsis ampullacea*.
