# Supplementary material for: Field Susceptibility of Almond (Prunus dulcis) Cultivars to Red Leaf Blotch Caused by Polystigma amygdalinum in Apulia (Italy) and Influence of Environmental Conditions
Source: Plants (Basel). 2026 Jan 7;15(2):188. doi: 10.3390/plants15020188 (PMC12845356; doi:10.3390/plants15020188)
Supplement: Supplementary file 1 [file plants-15-00188-s001.zip › plants-4037477-supplementary.pdf]

plants

Supplementary Materials: The following supporting information can be downloaded at: https://www.mdpi.com/article/doi/s1.

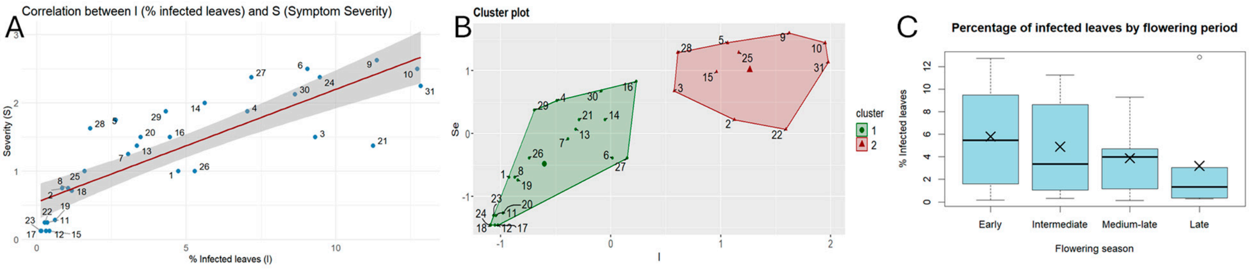

Figure S1: (A) Correlation between incidence (I) and severity (Se) of Red Leaf Blotch (RLB). Each point represents the average incidence and severity values for one of the 31 almond cultivars grown in Field 1. (B) Cluster analysis of almond cultivars based on I and Se values recorded in 2024. The optimal number of clusters was determined using the NbClust algorithm, and clustering was performed using the k-means method. Cluster 1 (green) corresponds to less susceptible cultivars, whereas Cluster 2 (red) comprises more susceptible cultivars. Cultivar identification numbers correspond to those listed in Table 1. (C) Percentage of infected leaves across flowering-period categories (early, intermediate, medium–late, late). Each boxplot shows the distribution of infection levels within each flowering group; the horizontal line indicates the median, and the "X" denotes the mean.

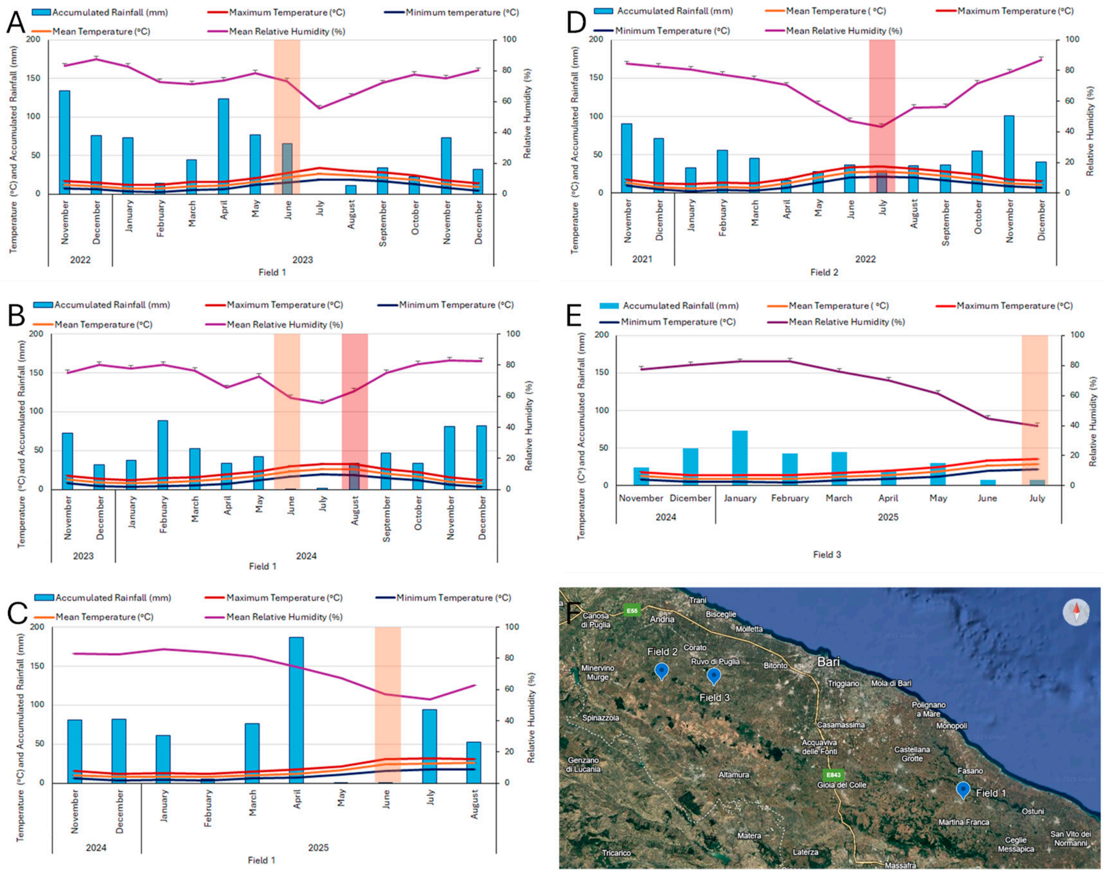

Figure S2. A-C) Meteorological data collected into the Field 1 (ex situ biodiversity germplasm collection experimental field at the Centro di Ricerca, Sperimentazione e Formazione “Basile Caramia”, Locorotondo BA, Apulia, Italy, 40°45'27.2"N 17°20'22.3"E; years 2023-2024-2025; meteorological station (Neetra IoT Agrismart ID Centralina C6C240 Label C6C240, 40°45'24.5"N 17°20'27.7"E); D) Field 2 (commercial orchard, Corato, BA, Apulia, Italy, 41°03'45.7"N 16°18'06.5"E, year 2021-2022); E) Field 3 (commercial orchard, Ruvo di Puglia, BA, Apulia, Italy, 41°03'02.9"N 16°28'55.2"E, year 2024 - 2025); for Fields 2 and 3, the meteorological data were extracted from NASA Prediction Of Worldwide Energy Resources [33]. F) Map of the Apulia region with the geolocation of the Fields under study.

Table S1. Details on the primer sets used.

**Target genomic region^**	**Name**	**Sequence (5'-3')**	**Product size (bp)**	**Reference**
ITS2	PamyI2F4	GAAGTCCAATCAAGCCGTAG	99	[29]
	PamyI2R2	GTTTCACTACGCTCAGAGTC		
ITS1-5.8S-ITS2	PyITS1	TCCGTTGGTGAACCAGCGG	560	[4]
	ITS4	TCCTCCGCTTATTGATATGC		

^The primer pair PamyI2F4/I2R2 was used in qPCR, while the pair PyITS1/ITS4 was used in end-point PCR.

Table S2. GenBank references sequences used for phylogenetic analysis.

**Species**	**Strain number**	**Country, host**	**GenBank/EBI accession number**
Polystigma amygdalinum	EA1	Iran, *Prunus dulcis*	KC756360
Polystigma amygdalinum	HB3-550	Iran, *Prunus* sp.	JQ995323
Polystigma amygdalinum	K1	Iran, *Prunus dulcis*	KC756361
Polystigma amygdalinum	KARE2767	USA, nr^	PV491257
Polystigma amygdalinum	KARE2768	USA, nr	PV491258
Polystigma amygdalinum	KARE2771	USA, nr	PV491259
Polystigma amygdalinum	KARE2773	USA, nr	PV491260
Polystigma amygdalinum	M4	Iran, *Prunus dulcis*	KC756362
Polystigma amygdalinum	MA1	Iran, *Prunus dulcis*	KC756363
Polystigma amygdalinum	MA2	Iran, *Prunus dulcis*	KC756364
Polystigma amygdalinum	MM1	Iran, *Prunus dulcis*	KC756365
Polystigma amygdalinum	TO1	Spain, *Prunus dulcis*	MH205935
Polystigma amygdalinum	TO10	Spain, *Prunus dulcis*	MH205939
Polystigma amygdalinum	TO15	Spain, *Prunus dulcis*	MH205940
Polystigma amygdalinum	TO16	Spain, *Prunus dulcis*	MH205941
Polystigma amygdalinum	TO18	Spain, *Prunus dulcis*	MH205942
Polystigma amygdalinum	TO5	Spain, *Prunus dulcis*	MH205936

Polystigma amygdalinum	TO7	Spain, *Prunus dulcis*	MH205937
Polystigma amygdalinum	TO9	Spain, *Prunus dulcis*	MH205938
Polystigma amygdalinum	Y1	Iran, *Prunus dulcis*	KC756366
Polystigma fulvum	MFLU 18-0261	Belarus, *Prunus padus*	MK429738
Polystigma pusillum	MM 19	Panama, *Andira inermis*	KX451899
Polystigma rubrum	MFLU 15-3091	Russia, *Poaceae*	KY594023
Polystigma rubrum	MFLU 18-0270	Russia, *Prunus domestica*	MK429747
Polystigma rubrum	MFLU 18-0271	Russia, *Prunus stepposa*	MK429748
Pseudopestalotiopsis ampullacea	NKT0P06	India, *Camellia sinensis*	PQ569990

^nr: not reported.
